# Binding of canonical Wnt ligands to their receptor complexes occurs in ordered plasma membrane environments

**DOI:** 10.1111/febs.14139

**Published:** 2017-07-06

**Authors:** Erdinc Sezgin, Yagmur Azbazdar, Xue W. Ng, Cathleen Teh, Kai Simons, Gilbert Weidinger, Thorsten Wohland, Christian Eggeling, Gunes Ozhan

**Affiliations:** ^1^ MRC Human Immunology Unit Weatherall Institute of Molecular Medicine University of Oxford UK; ^2^ Izmir International Biomedicine and Genome Institute (iBG‐izmir) Dokuz Eylul University Izmir Turkey; ^3^ Department of Medical Biology and Genetics Dokuz Eylul University Medical School Izmir Turkey; ^4^ Department of Chemistry and Center for BioImaging Sciences National University of Singapore Singapore; ^5^ Institute of Molecular and Cell Biology Agency for Science, Technology and Research Singapore Singapore; ^6^ Max Planck Institute of Cell Biology and Genetics Dresden Germany; ^7^ Institute of Biochemistry and Molecular Biology Ulm University Germany

**Keywords:** canonical Wnt, lipid raft, plasma membrane lipid, membrane trafficking, Wnt/β‐catenin pathway

## Abstract

While the cytosolic events of Wnt/β‐catenin signaling (canonical Wnt signaling) pathway have been widely studied, only little is known about the molecular mechanisms involved in Wnt binding to its receptors at the plasma membrane. Here, we reveal the influence of the immediate plasma membrane environment on the canonical Wnt–receptor interaction. While the receptors are distributed both in ordered and disordered environments, Wnt binding to its receptors selectively occurs in more ordered membrane environments which appear to cointernalize with the Wnt‐receptor complex. Moreover, Wnt/β‐catenin signaling is significantly reduced when the membrane order is disturbed by specific inhibitors of certain lipids that prefer to localize at the ordered environments. Similarly, a reduction in Wnt signaling activity is observed in Niemann–Pick Type C disease cells where trafficking of ordered membrane lipid components to the plasma membrane is genetically impaired. We thus conclude that ordered plasma membrane environments are essential for binding of canonical Wnts to their receptor complexes and downstream signaling activity.

**Abbreviations**
ACF: autocorrelation function
CM: conditioned media
COase: cholesterol oxidase
DMEM: Dulbecco's modified Eagle medium
DRM: detergent‐resistant membrane
FCS: fluorescence correlation spectroscopy
GM1: monosialotetrahexosylganglioside
GP: generalized polarization
NPC: Niemann–Pick Type C
Rec: recombinant
SL2: S‐laurdan2
SPIM: single plane illumination microscopy

## Introduction

Wnt/β‐catenin signaling, also referred to as the canonical Wnt pathway, plays pivotal roles in regulation of embryonic development, maintenance of tissue homeostasis and regeneration 1, 2, 3, 4, 5. Mutations in Wnt signaling components and modulators are thus associated with various genetic disorders, cancers, and degenerative diseases 6. Development of new therapeutic approaches for these diseases necessitates elucidation of the molecular mechanisms underlying fine‐tuning of the pathway at various levels. This fine‐tuning can be based on a variety of events including ligand–receptor complex formation, phosphorylation by kinases, adjustment of intracellular β‐catenin levels, and tissue‐specific modulator activities 7, 8, 9, 10.

In the absence of an active canonical Wnt ligand, β‐catenin is phosphorylated in the cytoplasm by a complex of proteins, the so‐called destruction complex, which includes the serine–threonine kinases Glycogen synthase kinase 3 (Gsk3) and Casein kinase 1 (Ck1), the scaffold protein Axin and the tumor suppressor Adenomatous polyposis coli (APC), leading to proteasomal degradation of β‐catenin and suppression of Wnt/β‐catenin signaling 11. β‐Catenin signaling is triggered by the binding of a canonical Wnt ligand to a Frizzled (Fz) receptor and the coreceptor low‐density lipoprotein receptor‐related protein 6 (Lrp6) or its close relative Lrp5 12. Wnt binding leads to the phosphorylation and endocytosis of Lrp5/6, which are both essential events for pathway activation 13, 14, 15. Another key determinant in pathway activation is the stability of cytoplasmic β‐catenin. Lrp5/6 phosphorylation by Gsk3 and Ck1 leads to the recruitment of the cytoplasmic proteins, Disheveled (Dvl) and Axin, to the receptor complex, which in turn inhibits the destruction complex, allowing stabilized β‐catenin to translocate into the nucleus and bind to the transcription factors of the lymphoid enhancer‐binding factor (Lef) and T‐cell factor (Tcf) family to activate target gene transcription 12, 16.

While the downstream events triggered by receptor activation have been widely studied, little is known about the molecular processes that are key for Wnt ligand–receptor interactions. Specifically, hardly anything has been revealed with respect to the influence of the receptors’ lipid membrane environment on Wnt pathway activation. We and others have shown that the coreceptor Lrp6 and a membrane‐bound pathway modulator Lypd6 tend to localize differently at the plasma membrane, with the former being distributed throughout the membrane and the latter becoming localized to ordered membrane domains 9, 13, 17. Ordered membrane domains, often generalized as membrane (lipid) rafts, are specialized membrane environments or (nano‐) domains that are distinguished as relatively more organized (or ordered) and are characterized by dynamic assemblies of saturated lipids, sterols, and lipid‐anchored proteins 18, 19. They provide a platform for compartmentalizing and modulating several cellular processes including receptor clustering and regulation of signal transduction 19, 20, 21, 22, 23, 24. Stimulated Toll‐like receptors (TLRs), for instance, cluster in lipid rafts and induce protumor and antitumor responses 25, 26. Upon phosphorylation, the hematopoietic protein tyrosine phosphatase is likewise targeted to lipid rafts and interferes with T‐cell receptor signaling 27. However, little is known about the mechanistic roles of these ordered environments in regulation of Wnt signaling pathways. The impact of membrane organization on Wnt/β‐catenin signaling has partially been uncovered for Lrp6 activation after Wnt ligand binding. While Lrp6 is rather evenly distributed at the plasma membrane, its phosphorylation in response to Wnt is specifically detected in the ordered membrane environments 13, 17. This compartment‐specific phosphorylation of Lrp6 is ensured by a glycophosphatidylinositol‐anchored protein called Lypd6, which also partitions into the ordered membrane environments and acts as a positive regulator of Wnt/β‐catenin signaling 9. Two possibilities could then explain specific pathway activation in the ordered membrane domains: either (a) Wnt ligands bind to Fz and Lrp6 receptors, which are distributed throughout the plasma membrane, but ternary complexes of Wnt, Fz, and Lrp6 can only form in the ordered domains, for example, due to the specific localization of other cofactors like Lypd6 there; or (b) binding of Wnt ligands to their receptors could already preferentially occur in the ordered membrane domains. However, it is not clear whether there is an environment preference for the binding of the Wnt ligand to its receptors and whether the immediate membrane environment of the receptors is critical for the signaling activity.

Here, we addressed the influence of the membrane environment of the receptor complex on binding and signaling activity of the canonical Wnt. Specifically, we investigated the membrane order preference of canonical Wnt ligands (Wnt3, Wnt3a, and Wnt8a) and the proteins of the receptor complex (Fz8, Lrp6, and Lypd6) in HEK293T cells. Using detergent resistance assay, we showed that while Wnt ligands were preferably present at the ordered environments, Fz8 and Lrp6 were distributed both in the ordered and disordered parts, indicating that Wnt binding to its receptor complex occurs preferentially in ordered membrane environments. Single molecule–sensitive experiments with SPIM‐FCS, [single plane illumination microscopy (SPIM) combined with fluorescence correlation spectroscopy (FCS)] indicated that Wnt ligand undergoes domain‐like diffusion that is dependent on ordered membrane lipids. This was further supported by the observation that the general lipid order decreased in the plasma membrane after ligand binding, likely due to endocytosis of the Wnt‐receptor complex. Most importantly, disruption of the ordered membrane environments in mammalian cells and zebrafish embryos dramatically reduced Wnt/β‐catenin signaling. Similarly, Wnt signaling activity appeared to be reduced in Niemann–Pick Type C (NPC) disease cells where cholesterol as well as sphingomyelin trafficking to the plasma membrane is hampered due to the mutations in NPC genes 28. Restoring these lipids in NPC disease cells increased Wnt signaling activity close to the wild‐type cells. Hence, we propose that initial interaction of the canonical Wnt ligand with its receptors preferentially occurs in the ordered environments of the plasma membrane and that this segregation of Wnt is essential for the generation and transmission of an active signal to the interior of the cell.

## Results

### Partitioning of Wnt‐receptor complex elements in plasma membrane environments

**Figure 1:**
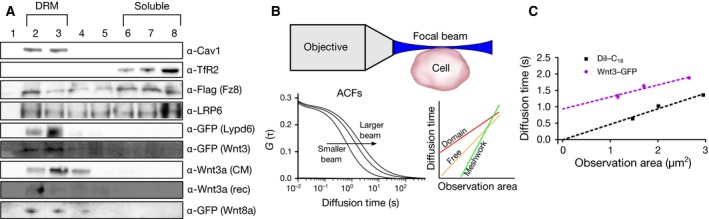
Membrane order preference of the canonical Wnt ligand and its receptors. (A) Western blot of detergent extracts of the plasma membrane for Fz8 (detected by its Flag tag), Lrp6, Lypd6 (detected by its GFP tag), Wnt3 (detected by its GFP tag), Wnt3a [from conditioned media (CM)], recombinant Wnt3a (Wnt3a rec), Wnt8a, and the controls caveolin1 (Cav1 with known detergent‐resistant fraction preference) and transferrin‐receptor (TfR2 with known detergent soluble fraction preference) with DRM and soluble positions marked. (B) A schematic of SPIM‐FCS measurements. A focal volume is formed with a single plane illumination. Signal is recorded from the apical membrane of the cells. Later, the signal is correlated with different bin sizes to form varying sizes of observation areas. With larger area, the diffusion time is higher while with a smaller area, the diffusion time is lower. The extent of this change depends on the diffusion mode of the molecule, whether it is free or hindered. Dependence of diffusion time to observation area is shown for different diffusion modes; domain, free, and meshwork diffusion. (C) Wnt3 undergoes domain‐like diffusion unlike DiI‐C_18_ (used as a control for free diffusion). Statistical significance between the two treatments is determined by Kolmogorov–Smirnov (KS) test statistics. Error bars represent the standard error of the mean at each binned area (number of data points is 36 for 1 × 1 binning, 25 for 2 × 2 binning, and 16 for 3 × 3 binning).

To figure out the direct impact of membrane environment on canonical Wnt signaling, we first aimed to determine the localization of the ligand–receptor complex components of Wnt/β‐catenin signaling within the plasma membrane of live HEK293T cells. On the one hand, we employed a well‐established biochemical assay, where cellular membranes are dissolved with detergents and isolated detergent‐resistant membrane (DRM) fractions are considered as an indicator of ordered membrane environments 29, 30. By fractionation of DRMs from cell extracts via flotation, we detected endogenous Lrp6 throughout the DRMs and soluble membrane fractions, while Lypd6 was predominantly in the DRMs (Fig. 1A), as others and we have previously shown 9, 13, 17. We found that the previously untested receptor Fz8 was also almost evenly distributed between DRMs and soluble fractions with slightly increased abundance in the soluble ones (Fig. 1A). Intriguingly, Wnt3a ligand, known to activate canonical Wnt signaling and here supplied as conditioned media (Wnt3a CM) was solely detected in the DRMs, indicating that canonical Wnt binding to its receptors exclusively occurs in ordered membrane environments (Fig. 1A). We tested the recombinant Wnt3a (Wnt3a rec) protein as well as two other canonical Wnt ligands, Wnt3 and Wnt8a, all of which partitioned similarly into DRMs (Fig. 1A). Although DRM partitioning is useful to demonstrate the propensity of membrane molecules to partition into the ordered membrane domains, it may not accurately reflect the native behavior of molecules due to the harsh extraction conditions, particularly the cold temperatures needed for extraction. Thus, we set out to validate our findings by applying a single molecule–sensitive technique; single plane illumination microscopy (SPIM) combined with fluorescence correlation spectroscopy (FCS). SPIM‐FCS is a camera‐based imaging FCS modality 31 that couples SPIM with fast array detectors such as electron‐multiplying charged coupled device (EMCCD) cameras 32. The FCS diffusion law 33 provides information on the nano‐scale diffusion behavior of molecules by utilizing dependence of the diffusion coefficient of the molecule to the observation spot. If molecules undergo free Brownian motion, their diffusion coefficient is constant with varying observation area. However, molecules in the plasma membrane undergo various types of hindered (nonfree) diffusion 33. In domain‐like diffusion, for instance, the diffusion coefficient decreases with decreasing observation spot 34. SPIM‐FCS has the capacity to perform binning of pixels after acquisition, thus, creating multiple observation areas from a single FCS measurement (Fig. 1B) 35, 36. Here, we applied SPIM‐FCS to observe the nanoscale diffusion of Wnt3 ligand. To this end, we expressed zebrafish Wnt3‐EGFP in zebrafish embryos. SPIM‐FCS data showed that Wnt3‐EGFP undergoes domain‐like diffusion in embryos, unlike DiI‐C_18_ dye known to diffuse freely in the membrane (Fig. 1C) 37. DRM flotation and SPIM‐FCS data together strongly suggest that Wnt ligand preferably binds to the pool of Fz8 and Lrp6 receptors that reside in the ordered membrane environments and undergoes domain‐like diffusion.

### Canonical Wnt stimulation reduces the order of the plasma membrane

**Figure 2:**
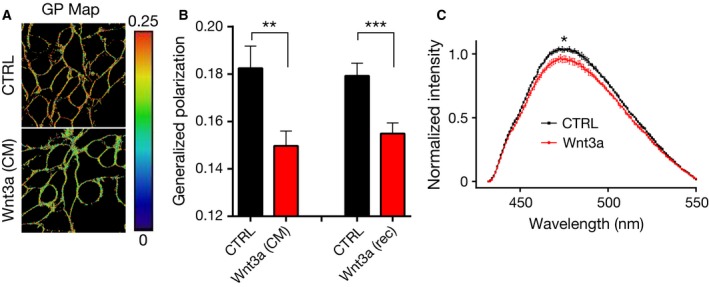
Plasma membrane order is altered upon canonical Wnt stimulation. (A) Representative generalized polarization (GP) maps in the plasma membrane of HEK293T cells treated with either control‐ (top) or Wnt3a‐conditioned media (CM) (bottom) using the polarity sensitive dye Di‐4‐AN(F)EPPTEA and confocal microscopy in combination with spectral detection (large GP values indicate high membrane ordering; red in the color scale). (B) Average and standard deviation (error bars) of GP values determined from 10 images (each image contained multiple cells) for both Wnt3a CM and Wnt3a rec. (C) Average and standard deviation (error bars, three independent experiments) of fluorescence emission spectra of SL2 in HEK293T cells treated with either control‐conditioned (black) or Wnt3a‐conditioned (red) media (for panel C, *P* value was determined using the intensity at λ = 475 nm). Statistical significance was determined using unpaired *t‐*test. ***P < 0.001, **P < 0.01, *P < 0.05.

Since our data indicate that Wnt binding to its receptors preferably takes place in the ordered membrane environments, we next investigated whether Wnt‐receptor complex formation influences the order of the plasma membrane environments. To this end, we measured the order of the plasma membrane in live HEK293T cells after labeling with the polarity‐sensitive dye Di‐4‐AN(F)EPPTEA 38. This dye changes its emission spectrum depending on the membrane order or lipid packing 39. Using spectral imaging on a confocal microscope 40, we calculated values of generalized polarization (GP) from the spectrum of fluorescence emission; the higher the GP values, the higher the membrane order is (see Materials and methods). We observed lower GP values in HEK293T cells treated with either with Wnt3a CM or Wnt3a rec than in control cells that were not stimulated with the ligand, strongly suggesting that the molecular order of the plasma membrane decreases upon Wnt stimulation (Fig. 2A,B). To confirm this, we exploited another membrane‐embedded polarity‐sensitive probe, SL2 41, which alters its fluorescence emission intensity (instead of emission spectrum) depending on the ordering of the membrane environment (lower orders result in loss of intensity). By analyzing Wnt3a‐ and control‐conditioned media‐treated HEK293T cells with fluorescence spectrophotometry, we observed a decrease in fluorescence intensity in Wnt3a‐treated cells compared to control cells, that is, the plasma membrane becomes less ordered upon Wnt stimulation (Fig. 2C). Thus, canonical Wnt stimulation of cells leads to a reduction in their plasma membrane order, most likely due to the fact that the relatively more ordered portions of the membrane are cointernalized with the receptor complex, leaving the noninternalized membrane less ordered.

### Membrane order is essential for canonical Wnt signaling activity

**Figure 3:**
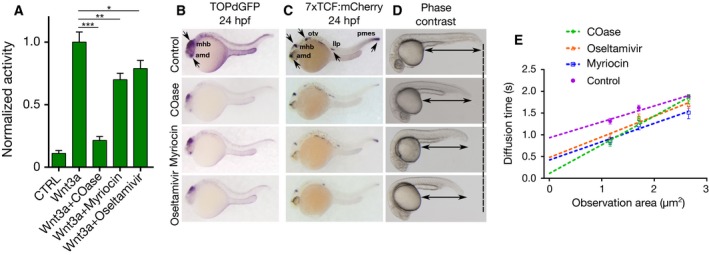
Ordered membrane environments are essential for Wnt3a‐mediated β‐catenin signaling. (A) Average and standard deviation (error bars, three independent experiments) values of pBAR luciferase reporter activity monitoring Wnt/β‐catenin signaling activity (normalized to renilla luciferase activity) in HEK293T cells treated with COase, myriocin, or oseltamivir and induced with the control‐ or Wnt3a‐conditioned media. (B, C) *GFP* or *mCherry* whole‐mount *in situ* hybridization showing downregulation of signaling in the two transgenic zebrafish embryos *TOP*dGFP (B) and *7xTcf*:mCherry (C) after treatment with COase, myriocin, and oseltamivir. Arrows highlight the regions where reduction in Wnt/β‐catenin signaling activity is observed. All expression domains detected in the control embryos are downregulated after drug treatment. Note that the drugs appear to influence these domains differently. amd, anterior‐most domain; mhb, midbrain–hindbrain boundary; otv, otic vesicle; llp, lateral line primordium; pmes, posterior mesoderm. (D) Morphological phenotypes at 24 hpf of embryos treated with cholesterol oxidase (3 U·mL^−1^, 23/24 embryos), myriocin (187.5 μm, 19/21 embryos), or oseltamivir (150 μm, 19/19 embryos) for 19 h. Arrows and the dashed line show the reduction of size in trunk and tail. (E) FCS diffusion law of Wnt3‐EGFP in live transgenic embryos treated with COase, myriocin, or oseltamivir. COase completely diminishes the domain diffusion while myriocin or oseltamivir significantly reduces it. Error bars represent the standard error of the mean at each binned area (number of data points is 36 for 1 × 1 binning, 25 for 2 × 2 binning, and 16 for 3 × 3 binning). Statistical significance is evaluated by unpaired *t*‐test for luciferase assay (panel A) and by a Kolmogorov‐Smirnov (KS) test, for SPIM‐FCS diffusion data (panel E). ***P < 0.001, **P < 0.01, *P < 0.05.

To test whether direct alteration of the membrane order influences Wnt/β‐catenin signaling activity, we specifically disturbed the ordered plasma membrane environments. Using the enzymes or drugs cholesterol oxidase (COase), myriocin, or oseltamivir, we depleted molecules that have previously been shown to be associated with the ordered membrane domains: cholesterol (by COase), sphingomyelin (by myriocin), or GM1 ganglioside (by oseltamivir). In all cases, we observed a significant decrease in Wnt/β‐catenin signaling in HEK293T cells, as confirmed by the activation of the pBAR reporter of Tcf/Lef‐mediated transcription (Fig. 3A).

To test these findings *in vivo*, we used embryos of two different transgenic zebrafish reporters of Wnt/β‐catenin signaling (Tg(*TOP*:dGFP)^w25^ 42 and Tg(*7xTcf‐Xla.Siam*:nlsm‐Cherry^ia^) 43), and asked whether the pathway activity was affected after treatment by the above‐mentioned enzymes/drugs (COase, myriocin, or oseltamivir). Enzyme/drug treatments, similarly to HEK293T cells, decreased the signaling activity in zebrafish embryos as detected by decreased reporter expression at 24 hpf (Fig. 3B,C). To test whether the lipids associated with the ordered membrane domains are also necessary for activation of Wnt/β‐catenin signaling during early development, we treated the embryos with the aforementioned enzymes/drugs at gastrulation (5 hpf, 30% epiboly). We found that depletion of each lipid at gastrulation caused reduction in the size of trunk and tail, reminiscent of the morphological effect produced by the knockdown of *wnt8* (Fig. 3D). Thus, we conclude that the ordered environments in plasma membrane are essential for the transmission of signal generated by canonical Wnt‐receptor complex to downstream signaling elements and consequential pathway activation.

We also tested whether the enzymes/drugs that disrupt ordered membrane environments affect the domain‐like diffusion of Wnt3. We performed SPIM‐FCS measurements on enzyme/drug‐treated live double transgenic zebrafish embryos from in‐cross of Tg(‐4.0*wnt3*:Wnt3EGFP)^F2^ and Tg(‐4.0*wnt3*:Wnt3EGFP)^F3^. Domain‐like diffusion was almost completely abolished in COase‐treated embryos, leading to free diffusion (Fig. 3E). In myriocin‐ and oseltamivir‐treated embryos, domain‐confined diffusion was reduced and inclined significantly, although not completely, toward free diffusion (Fig. 3E). Thus, we conclude that the domain‐like diffusion of Wnt3 is dependent on ordered membrane lipids and is necessary for the signaling activity.

### Wnt/β‐catenin signaling is reduced in membrane cholesterol‐deficient Niemann–Pick C disease cells

**Figure 4:**
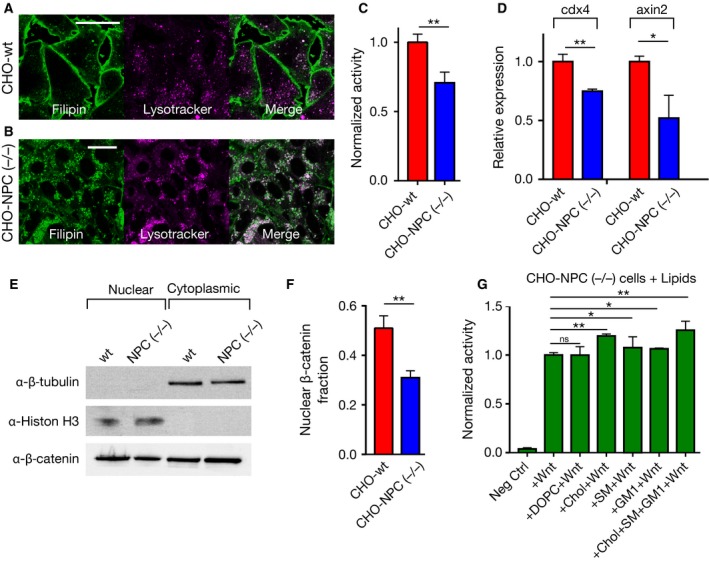
Wnt/β‐catenin signaling in Niemann–Pick Type C (NPC) cells. Cholesterol distribution in (A) CHO‐wt cells and (B) CHO‐NPC (−/−) cells. Filipin (green) labels cholesterol, lysotracker (magenta) labels lysosomes. Scale bars are 20 μm. (C) Wnt/β‐catenin signaling is reduced in CHO NPC (−/−) cells compared to CHO‐wt cells. (D) *cdx4* and *axin2* expression levels determined by qRT‐PCR in CHO‐NPC (−/−) cells shown relative to those in CHO‐wt cells. (E) β‐catenin localization in nuclear (marked by Histon H3) and cytoplasmic (marked by β‐tubulin) portions in CHO‐wt and CHO‐NPC (−/−) cells and (F) quantitation of this localization. Nuclear β‐catenin fraction in CHO‐NPC (−/−) cells is reduced (0.3) as compared to CHO‐wt cells (0.5). (G) Wnt/β‐catenin signaling activity in CHO‐NPC (−/−) cells fed with cholesterol, DOPC, sphingomyelin, and GM1. DOPC does not alter the signaling capacity of cells while cholesterol, sphingomyelin, GM1 and a mixture of these three increase the signaling. Statistical significance was evaluated by unpaired *t*‐test. Error bars are standard deviations of three independent experiments. **P < 0.01, *P < 0.05.

To examine the effect of membrane order on Wnt/β‐catenin signaling activity in the context of a lipid storage disorder, we exploited the disease model of Niemann–Pick disease type C (NPC), which is a rare progressive genetic disorder characterized by an inability of the body in trafficking of mainly cholesterol within the cells and consequent accumulation of cholesterol in the lysosomal or late endosomal compartments 28, 44. As cholesterol is not properly targeted to the plasma membrane, it becomes trapped inside the lysosomal compartments, resulting in the overall reduction of the membrane cholesterol. To confirm that cholesterol trafficking in these cells is perturbed, we stained wild‐type Chinese hamster ovary (CHO‐wt) cells with filipin, an inherently fluorescent cholesterol‐binding molecule 45. We observed that most of the filipin was localized at the plasma membrane in CHO‐wt cells (Fig. 4A), while in CHO cells where NPC genes are knocked out (CHO‐NPC (−/−)) 46 it colocalized with lysotracker, a fluorescent probe that labels the lysosomes in cells (Fig. 4B). Cholesterol and other lipids (such as sphingomyelin) that become trapped in these intracellular compartments in NPC disease 28, 47 are mainly ordered domain lipids 46. Therefore, reduced amount of cholesterol in CHO‐NPC (−/−) cells is likely to disturb dominantly their ordered membrane environments. By measuring pBAR activation after Wnt3a CM stimulation, we found that Wnt/β‐catenin signaling was significantly reduced in CHO‐NPC (−/−) cells compared to CHO‐wt cells (Fig. 4C). Knockout of NPC genes also caused a reduction in expression of the direct Wnt/β‐catenin target genes *axin2* and *cdx4* 48, 49 (Fig. 4D) as well as a decrease in nuclear β‐catenin localization (Fig. 4E,F), both confirming the reduced Wnt/β‐catenin signaling activity in CHO‐NPC (−/−) cells. These results are consistent with our previous finding that the ordered membrane environment component cholesterol is necessary for Wnt/β‐catenin signaling. Besides cholesterol, trafficking of sphingomyelin and gangliosides, two other ordered membrane lipids we tested, is also disturbed in CHO‐NPC(−/−) cells 28, thus the decreased Wnt activity in these cells is likely due to a combined effect of reduced levels of these lipids. We tested this hypothesis, by replenishing CHO‐NPC (−/−) cells with these lipids. First, as a control we fed the cells with an unsaturated lipid [1,2‐dioleoyl‐sn‐glycero‐3‐phosphocholine (DOPC)] and observed no notable change in pBAR activation in these cells (Fig. 4G). While addition of sphingomyelin and GM1 enhanced the reporter activity significantly, a higher increase was observed when the cells were fed with cholesterol. A mixture of cholesterol, sphingomyelin, and GM1 showed the most remarkable effect (Fig. 4G) and increased the reporter activity to a similar level of CHO‐wt cells (compare with Fig. 4C). These results indicate that Wnt/β‐catenin signaling activity is reduced in NPC disorder and that this activity can be restored by replenishing the cells with lipids of ordered membrane environments.

## Discussion

The events of Wnt/β‐catenin signaling that occur downstream of the Wnt‐receptor complex formation have been thoroughly disclosed. However, little is known about how the initial interactions between Wnt and its receptors are mediated at the plasma membrane. Our study addresses the influence of membrane lateral heterogeneity on canonical Wnt‐receptor interaction and subsequent β‐catenin pathway activation. Our biochemical and biophysical data suggest that binding of the canonical Wnt ligand to its receptors Fz8 and Lrp6 predominantly occurs in ordered environments of the plasma membrane (often referred to as lipid rafts), although Fz8 and Lrp6 are evenly distributed across ordered and disordered membrane regions. This indicates that Wnt binding to its receptor complex is a selective process and that the canonical Wnt ligands predominantly recognize the pool of receptors residing in the ordered membrane environments. This selective feature correlates with the signaling activity (both *in vitro* and *in vivo*), that is, Wnt/β‐catenin signaling is only active in the presence of ordered membrane environments and diminishes if those are depleted, for example, chemically by treatment with certain lipid‐targeting enzymes/drugs or genetically as in the case of CHO‐NPC(−/−) cells. Our data thus suggest that, for the canonical Wnt receptors (Lrp6 and Fz8) to be functional, they have to reside in the ordered membrane environments (e.g., due to potentially optimized conformational states or lipid‐assisted Wnt binding), while their enrichment in the ordered domains seems to be of less importance. Our study elucidates a novel critical step in pathway activation by revealing the necessity of the ordered plasma membrane environments during initial binding of canonical Wnt to its receptors. These results point out a novel activation mode for the Wnt/β‐catenin (and possibly other) signaling pathway(s), suggesting that the ligand–receptor interaction is a process which is rendered possible only in particular nano‐environments within the plasma membrane and that this interaction is essential for compartmentalization and activation of cell signaling processes.

As deregulation of Wnt/β‐catenin signaling has been linked to numerous human diseases, the pathway components and modifiers that are able to modify and regulate signaling at various levels have been assessed as drug targets. Although a vast amount of compounds have been identified to alter Wnt signaling activity 50, 51, few therapeutic agents specifically inhibiting the pathway have only recently entered clinical trials 52, 53. Among those agents are the Wnt secretion inhibitors (that block the acyltransferase Porcupine), Fz‐targeting antibodies, Dvl inhibitors, inhibitors of tankyrases that stabilize Axin, and the Tcf‐β‐catenin complex antagonists 53, 54, 55, 56, 57, 58, 59. Owing to its pivotal role in many signaling events, the plasma membrane is frequently investigated for its proteins as drug targets 60, 61. Because certain membrane receptor proteins display preferential partitioning into ordered environments (or lipid rafts) at the membrane, the molecules that target these environments (also referred to as being raftophilic) could be potential drug candidates for targeting the compartmentalized activity of those signaling proteins. There is good evidence that the drug effectiveness is improved by particular modifications which increase their membrane affinity 62. Addition of a raftophilic lipid moiety, for instance, restrains the drug to the ordered environments and enables maintenance of high concentrations at the signaling site. For example, viral activity of HIV‐1 was dramatically inhibited by using a membrane‐anchored version of the HIV fusion inhibitor peptide T‐20 or by introducing a cholesterol anchor to another HIV‐1 fusion inhibitor that targets it to ordered membrane parts 63. Supporting evidence was obtained from studies on HBV infection, which was efficiently blocked by membrane anchoring via lipidation (myristoylation, stearoylation, or acylation) of the peptides derived from the viral surface glycoproteins 64, 65. Moreover, cholesterol appears to be a critical component of the membrane environments where Wnt binds to its receptors and more effective in pathway activation than sphingomyelin and GM1 gangliosides are. Thus, we believe that interactions between membrane lipid domains and the Wnt ligand may potentially be considered for the development of new drugs that interfere with signaling activity.

The NPC disease is caused by defective lipid trafficking (cholesterol, sphingolipids, and likely also gangliosides) and their consequent accumulation in lysosomes 28. The disease has so far been associated with the lysosomal accumulation of the lipids, but not with the altered membrane composition. Here, we show that vital signaling events such as Wnt/β‐catenin signaling that require a certain compositional integrity of the plasma membrane could be severely impaired by perturbations of the membrane composition. It would be quite interesting to test how membrane composition is altered in Wnt signaling‐related diseases.

Overall, our study demonstrates how bulk properties of the cell membrane can influence a vital signaling pathway through regulation of ligand–receptor binding, paving the way for further investigation of specific lipid involvement in Wnt signaling activation as well as molecular details of this specific binding. The outcome may potentially be useful for developing new strategies to increase target specificity of drugs that interfere with the Wnt‐receptor complex interactions at the plasma membrane. A potentially promising approach would be high‐end lipidomics technology to perform lipid fingerprinting on Wnt/β‐catenin signaling and characterize lipid compositions of the membrane environments where Wnt binds to its receptors to form an active signaling complex.

## Materials and methods

### Cell culture

The HEK293T cells were grown in Dulbecco's modified Eagle medium (DMEM) supplemented with 10% FBS. CHO‐wt and CHO‐NPC (−/−) cells were grown in DMEM F12 supplemented with 10% FBS. SH‐SY5Y cells obtained from ATCC (Manassas, VA, USA) were grown in DMEM supplemented with 10% FBS and 1% PS (penicillin and streptomycin) at 37 °C in 5% (v/v) CO_2_ humidified environment.

### Transgenic fish lines

Following transgenic zebrafish (*Danio rerio*) lines were outcrossed to wild‐type zebrafish and identified as described previously for Tg (*TOP*dGFP)^w25^ and Tg(*7xTcfF‐Xla.Siam*:nlsmCherry) 42, 43. These transgenic lines were used as reporters of Wnt/β‐catenin signaling activity. Double transgenic embryos from in‐cross of Tg(‐4.0*wnt3*:Wnt3EGFP)^F2^ and Tg(‐4.0*wnt3*:Wnt3EGFP)^F3^, which express functional fusion protein Wnt3‐EGFP in the brain, were screened to obtain appropriate Wnt3‐EGFP expression for SPIM‐FCS studies 66.

### Fluorescence staining of the cells

For DiI‐C_18_ staining (1,1′‐dioctadecyl‐3,3,3′,3′‐tetramethylindocarbocyanine perchlorate), stock DiI‐C_18_ prepared in dimethyl sulfoxide was diluted to a final concentration of 50 nm in 1 × Hanks’ balanced salt solution (HBSS). SH‐SY5Y cells were plated onto a 35‐mm dish containing custom‐cut No. 1 cover slips (0.13–0.16 mm thickness) and allowed to grow for 48 h. Cells were then stained and incubated with 50 nm DiI‐C_18_ in 1 × HBSS for 25 min at 37 °C in 5% (v/v) CO_2_ humidified environment. SH‐SY5Y cells were washed twice with 1 × HBSS and mounted into SPIM chamber containing 1 × HBSS as the imaging medium.

### Drug and enzyme treatment of zebrafish embryos and whole‐mount *in situ* hybridization

For organogenesis stage assay, zebrafish embryos at 10 h postfertilization (hpf) were incubated in the enzyme or chemical with the final concentrations of 2.4 U·mL^−1^ for cholesterol oxidase, 12.5 μm for myriocin, or 20 μm for oseltamivir for 14 h. For gastrula stage assay, embryos at 5 hpf were incubated in the enzyme or chemical with the final concentrations of 3 U·mL^−1^ for cholesterol oxidase, 187.5 μm for myriocin, or 150 μm for oseltamivir for 19 h. Enzyme/chemical was added onto the embryos held in E3 medium (5 mm NaCl, 0.17 mm KCl, 0.33 mm CaCl_2_, and 0.33 mm MgSO_4_, pH 6.8–6.9) in 24‐well plates. Embryos were fixed at 24 hpf in 4% paraformaldehyde containing PBS overnight and whole‐mount *in situ* hybridization (WMISH) was performed as described previously 67.

For SPIM‐FCS experiments on double transgenic embryos from in‐cross of Tg(‐4.0*wnt3*:Wnt3EGFP)^F2^ and Tg(‐4.0*wnt3*:Wnt3EGFP)^F3^, both transgenic adult zebrafish and embryos were maintained and generated at the zebrafish facility in the Institute of Molecular and Cell Biology (IMCB, Singapore). Briefly, 56 hpf zebrafish embryos were incubated with the respective chemicals/enzymes for 16–19 h at 28.5 °C. After treatment, embryos (72–75 hpf) were anesthetized with 0.05% (w/v) tricaine (Sigma‐Aldrich, St. Louis, MO, USA) dissolved in 1 × E3 medium for 30 min and subsequently mounted into a thin glass capillary tube with 1% low melting point agarose for SPIM‐FCS measurements in 1 × PBS containing 0.05% (w/v) of tricaine as the imaging medium.

### Subcellular fractionation and for β‐catenin localization

Cells were scraped into 1.5‐mL tubes and spun at 3000 rpm for 2 min. Supernatant was discarded and pellet was washed with 1 mL PBS. Cells were then lysed with 0.5 mL lysis buffer (50 mm Tris [pH = 7.5], 150 mm NaCl, %1 Triton X‐100, 10 mm NaF) that includes protease inhibitors. Lysates were incubated on ice for 10 min and spun down at 4 °C, 14000 rpm for 15 min after which the pellet (nuclei) and supernatant (cytoplasmic fraction) were collected separately.

### Quantitative PCR (qPCR)

The RNA was isolated using Trizol (Zymo Research, Irvine, CA, USA) and cDNA was synthesized with iScript reverse transcriptase (RT) (Biorad) using a 1 : 1 mixture of oligodT and random primers. (‐RT) controls were generated by replacing the iScript RT with water. qPCR was performed in triplicates with 1 : 10 diluted cDNA using a Go Taq qPCR master mix at Applied Biosystems (Foster City, CA, USA) 7500 Fast Real Time machine. Relative expression levels were determined after normalization to *rpl13a*. The following primers were used: hamster *cdx4*: 5′‐GGCCCCAACTTACCCACATT‐3′ and 5′‐GCTTGAGGGCAAGTCGTTCA‐3′; hamster *axin2*: 5′‐AGGTGTGGGGAAGGTCTTACA‐3′ and 5′‐TCTCAAGTATCCGACGCAGC‐3′; hamster *rpl13a*: 5′‐AAAAACGGATGGTGGTCCCT‐3′ and 5′‐CTTGGCCTTTTCCTTGCGTT‐3′.

### Wnt3a‐conditioned media (Wnt3a CM) production and recombinant Wnt3a (Wnt3a rec)

Wnt3a was produced from murine L cells. Cells were grown in a 10‐cm plate in DMEM supplemented with 10% FBS. When 90% confluence was reached, cells were diluted 1 : 10 and seeded in new 10‐cm plates. Wnt3a‐conditioned media were collected at 2, 4, and 6 days, aliquoted and stored at −20 °C until use. Recombinant Wnt3a was obtained from Abcam (ab81484, Cambridge, UK), dissolved in PBS supplemented with 0.25% BSA with 0.1 mg·mL^−1^ stock concentration and aliquoted for single use to avoid freeze/thaw cycles. Recombinant Wnt3a was added to the full growth media with 1 : 500 dilution (final concentration of 200 ng·mL^−1^). Cells were incubated in the Wnt3a‐enriched media for 6 h at 37 °C.

### Transfection and luciferase assay

Cells were seeded in triplicates on 24‐well plates and transfected with the firefly luciferase reporter pGL3 beta‐catenin‐activated reporter (pBAR 20 ng, 68), renilla luciferase reporter pGL4.73 hRLuc/SV40 (RLuc 5 ng; Promega, Madison, WI, USA), and a membrane GFP (75 ng as control for transfection) using Lipofectamine 2000 (Thermo Fisher Scientific, Waltham, MA, USA). The chemical/enzyme was added to the cells 24 h later and cells were incubated at 37 °C for the indicated durations as follows: oseltamivir (Cayman Chemicals, Ann Arbor, MI, USA) 20 μm for 48 h; myriocin 12.5 μm for 48 h; and cholesterol oxidase (Sigma‐Aldrich) 2.4 U·mL^−1^ for 12 h). Next, cells were stimulated with Wnt3a‐enriched media for 6 h. Reporter activity was measured with dual luciferase reporter assay kit (Promega). Significance was tested using Student's *t*‐test. ***P < 0.001, **P < 0.01, and *P < 0.05. Error bars represent standard deviation (SD).

### DRM flotation

The HEK293T cells were seeded in 10‐cm Petri dishes and transfected with Wnt3a or Wnt8a‐GFP 69. Two days after transfection, cells were washed with cold 1 × TNE buffer (50 mm Tris [pH 7.4], 150 mm NaCl, and 2 mm EDTA) and lysed. Cells were scraped with 1 × TNE containing phosphatase inhibitor (PI; Roche) and protease inhibitor cocktail (PIC; Sigma‐Aldrich) and centrifuged at 336 ***g*** for 5 min at 4 °C. Pellets were dissolved in 400 μL at 1 × TNE buffer containing PI, PIC, and 1% Triton X. Lysates were transferred to cold microfuge tubes and passed 20 times through a 26G needle. Samples were incubated on ice for 30 min and centrifuged at 3000 ***g*** for 5 min at 4 °C. Gradients were prepared by mixing 400 μL of supernatant with 800 μL of OptiPrep stock solution (60%; Sigma). The mixture was overlaid with 1680 μL of 30% OptiPrep in TNE and 720 μL of 5% Optiprep in TNE. Samples were ultracentrifuged at 226 800 ***g*** for 6 h using a type 90 Ti rotor in an Optima L‐100 XP ultracentrifuge and eight fractions of 400 μL were collected. Proteins were precipitated in 10% TCA for 15 min at −20 °C and centrifuged at 17 000 ***g*** for 1 h. Pellets were dissolved with sample loading dye and processed for western blotting. For Wnt3‐GFP, capped sense RNA was synthesized with mMessage mMachine Kit (Thermo Fisher Scientific) and injected into 1‐cell zebrafish embryos. Embryos were processed for detergent‐resistant membrane (DRM) flotation protocol at 7 hpf.

### Western blotting

Protein samples dissolved in sample loading dye were separated by SDS on 8% acrylamide‐bisacrylamide gel and transferred to polyvinylidene fluoride membrane (GE Healthcare Life science, Little Chalfont, UK). Blots were blocked in 5% milk powder for 1 h at room temperature (RT) and incubated with the following antibodies: rabbit anti‐GFP ((D5.1) XP^®^, 1 : 1000; Cell Signaling Technology) and rabbit anti‐Lrp6 (C5C7, 1 : 1000; Cell Signaling Technology, Danvers, MA, USA), ECL Rabbit IgG (HRP‐linked F(ab)2 fragment from donkey, 1 : 2500; GE Healthcare Bio‐Sciences), rabbit anti‐TfR2 (1 : 2000; Abcam) and mouse anti‐Caveolin1 (1 : 2000; BD Transduction Laboratories, Franklin Lakes, NJ, USA), mouse anti‐β‐ catenin (1 : 2500; BD Biosciences, Franklin Lakes, NJ, USA). Cellular localization kit (Cell Signaling) was used for Histon 3B and β‐tubulin (1 : 1000). Blots are quantified with imagej (ImageJ, U. S. National Institutes of Health, Bethesda, MD, USA) plugin (Analyze→Gels).

### Lipid‐packing measurements and spectral imaging of polarity‐sensitive dyes

Lipid packing of the Wnt3a‐enriched media (Wnt3a CM or Wnt3a rec)‐treated cells (2–6 h of stimulation) was done by using spectral imaging in combination with Di‐4‐AN(F)EPPTEA and SL2 dyes 38, 40. Cells were washed twice with phenol red‐free medium. Dyes were added onto cells in PBS with a final concentration of 2 μm and incubated for 5 min at RT. Cells were washed once with phenol red‐free medium before imaging. Spectral imaging was performed using a Zeiss 780 confocal laser‐scanning microscope in lambda mode. Di‐4‐AN(F)EPPTEA was excited at 488 and emission was collected between 500 and 700 nm. Generalized Polarization (GP) for Di‐4‐AN(F)EPPTEA was calculated using the formula below:

$$\text{GP}=\frac{I_{565}-I_{650}}{I_{565}+I_{650}}$$

where *I*_565_ and *I*_650_ are the fluorescence intensities at 565 and 650 nm, respectively. All calculations were done using freely available imagej plugin 40. Significance was tested using unpaired *t*‐test. *** indicates *P* < 0.001, **P < 0.01, and *P < 0.05. Error bars represent SD.

S‐laurdan2 intensity at 470 nm was used to assess lipid packing. Cuvette measurements of SL2 were performed using a fluorescence spectrophotometer. Cells were gently scraped off the Petri dishes and labeled with SL2 in PBS for 5 min at RT. Afterwards, cells were transferred into a cuvette and excited at 385 nm, emission was collected between 410 and 550 nm.

### SPIM‐FCS measurements

The SPIM‐FCS instrumentation was described previously 36. In the home‐built SPIM setup, two solid‐state diode pump lasers, 488 nm (OBIS 488 nm LX; Coherent Inc., Santa Clara, CA, USA) and 561 nm (LMX‐561S‐25‐COL‐PP; Oxxius S.A., Lannion, France), are used as excitation sources. Both lasers are beam expanded and directed to an achromatic cylindrical lens (*f* = 75 mm; Edmund Optics, Woodlands Loop, Singapore) to generate their respective light sheets. The light sheets are focused by overfilling the back focal plane of an illumination objective (SLMPLN 20×/0.25; Olympus, Singapore) to generate thin light sheets of thickness 1–1.2 μm. Subsequently, the light sheets are aligned at the focal plane of the detection objective (LUMPLFLN 60×/1.0W, WD = 2.0 mm; Olympus) placed perpendicular to the illumination objective. The samples were mounted on a motorized linear x‐, y‐, and z‐stage along with a rotation stage (XYZ‐linear stages: 3 × 8MT184‐13DC and rotation stage: 8MR174‐1‐20; Standa Ltd, Vilnius, Lithuania) into a custom‐made sample chamber. The water immersion detection objective is incorporated into the sample chamber through a mounting hole and mounted onto a piezo flexure objective scanner (P‐721 PIFOC; Physik Instruments, Sin Ming Ln, Singapore), which provides translation at nanometer precision. In the detection unit, the detection objective captures fluorescence emitted by the sample upon excitation by the appropriate light sheet. The 488‐nm light sheet was used to illuminate Wnt3‐EGFP transgenic zebrafish embryos while the 561‐nm light sheet excited DiI‐C_18_ stained SH‐SY5Y cells. A 488‐nm longpass filter (BLP01‐488R‐25; Semrock, Rochester, NY, USA) and a 561‐nm notch filter (NF561‐18; Thorlabs Inc., Newton, NJ, USA) are placed after the detection objective to reject scattered laser light. Thereafter, the fluorescence collected passes through an objective tube lens (LU074700, *f* = 180 mm; Olympus) and is split into two channels by a dual imaging optics unit (DV2, Photometrics, Tucson, AZ, USA; FF03‐525/50‐25 and LP02‐568RU‐25; Semrock) to select for the respective channels by the emission filters in the dual view unit. An EMCCD camera (Andor iXon3 860, 128 × 128 pixels; Andor Technology, Concord, MA, USA) was used as the detector where its chip is split into two channels that consist of 64 × 128 pixels each.

In SPIM‐FCS measurements, a stack of 50000–80000 images was acquired by the emccd camera software (Andor Solis for Imaging version 4.18.30004.0) at various regions of interest (ROIs). The camera was run at 500 and 1000 fps for Wnt3‐EGFP and DiI‐C_18_ measurements, respectively. After image acquisition, the data were analyzed by a home‐written plugin (Imaging_FCS 1.491, available at http://www.dbs.nus.edu.sg/lab/BFL/imfcs_image_j_plugin.html) for imagej (ImageJ). Autocorrelation analysis on the fluorescence fluctuations of each pixel was performed and the raw autocorrelation functions (ACF) were fitted with a SPIM‐FCS fitting model given in equation:

$$G_{\mathrm{SPIM}}\left(\tau \right)=\frac{1}{N}\cdot {\left\{\frac{\sqrt{4D\tau +\omega_{xy}^{2}}}{\sqrt{\pi }\cdot a}\cdot \left[e^{\left(-\frac{a^{2}}{4D\tau +\omega_{xy}^{2}}\right)}-1\right]+\mathrm{erf}\left(\frac{a}{\sqrt{4D\tau +\omega_{xy}^{2}}}\right)\right\}}^{2}+G_{\infty }.$$

In the equation, *G*_SPIM_(τ) is the ACF as a function of lag time (τ) for a two‐dimensional diffusion process determined by the plane illumination of SPIM and EMCCD detection 32, 35. *a* is the pixel side length in the object plane which is 400 nm in the setup. ω_xy_ is the lateral 1/e^2^ radii of the point spread function (PSF) of the maximum intensity (*I*_0_) at the focus of the observation volume. ω_xy_ of the system was calibrated using 100 nm fluorescent microspheres by FCS 70. The axial PSF, ω_z_, was determined before every experiment to ensure that measurements are conducted at the thinnest portion of the light sheet by fitting the light sheet width with a Gaussian function of 1/e^2^ radii. The number of particles (*N*), diffusion coefficient of the particle (*D*), and the convergence value of the ACF at infinity lag times (*G*_∞_) are the fit parameters. The χ^2^ value of the fit is used to evaluate the goodness of fit.

### FCS diffusion law in SPIM

The FCS diffusion law, first introduced by Wawrezinieck *et al*., provides information on subresolution membrane organizational features by measuring the dependence of the diffusion coefficient of membrane probes at various observation areas 33. The FCS diffusion law plot is generated by plotting the diffusion time (τ_D_) of a probe against observation area. For freely diffusing particles, τ_D_ scales linearly with observation area. Conversely, nonlinear transitions in the FCS diffusion law plots are observed for particles that undergo hindered diffusion due to confinement in membrane domains as well as particles that undergo hop diffusion as a result of meshwork compartmentalization in the cell membranes. However, these nonlinear transitions cannot be observed directly in experimental FCS diffusion law plots as the size of domains and meshes in the cell membranes are below the diffraction limit defined by the optical system. Therefore, we resolved this issue by extrapolating diffraction‐limited experimental FCS diffusion law plots to zero observation area to obtain the *y*‐intercept value (τ_0_). Positive, negative, and zero τ_0_ values indicate hindered diffusion with domain confinement, meshwork compartmentalized hop diffusion, and free diffusion, respectively. The magnitude of the positive τ_0_ value increases with domain density, domain size, and partitioning probability into domains. On the other hand, the magnitude of the negative τ_0_ value is affected by mesh size, density, and hop frequency of the particle. In SPIM‐FCS, various observation areas (*A*_eff_) can be defined by binning pixels postacquisition and convoluting the detection area (a) of a given bin size with the calibrated ω_xy_ of the SPIM system. The ACFs for each observation area is calculated and fitted with the equation above to obtain the diffusion time (τ_D_) of a given observation area. The SPIM‐FCS diffusion law is then plotted and fitted with the following empirical formula to obtain the τ_0_ value.

$$\tau_{D}\left(A_{\mathrm{eff}}\right)=\tau_{0}+\frac{A_{\mathrm{eff}}}{D_{\mathrm{eff}}}$$

The effective diffusion coefficient *D*_eff_ is defined as the inverse of the slope of the FCS diffusion law plot. SPIM‐FCS diffusion law was also analyzed by the imagej plugin ‘Imaging_FCS 1.491’.

## Conflict of Interests

No competing interests declared.

## Author contributions

ES and GO designed the experiments. ES, YA, XWN, and GO performed the experiments. KS, GW, CT, and TW contributed reagents, materials analysis tools. ES, CE, XWN, TW, and GO wrote the manuscript. All authors contributed to the discussion.
